# Identification of the blasting vibration characteristics of groundwater-sealed tunnel

**DOI:** 10.1038/s41598-023-40728-y

**Published:** 2023-08-21

**Authors:** Xiaokang Rao, Shengxiang Huang

**Affiliations:** School of Geodesy and Geomatics, Wuhan University, Wuhan, 430079 China

**Keywords:** Civil engineering, Sedimentology, Geology, Natural hazards, Applied physics

## Abstract

Blasting is widely used in mining, subway, demolition and groundwater-sealed tunnel, among them, the last one is widely concerned because of its many adjacent tunnels, high anti-seepage requirements, strict blasting control, etc. The identification of blasting characteristics is of great significance to the blasting construction and the safety evaluation of the groundwater-sealed tunnel. In view of the problem that conventional feature identification methods are less explored in groundwater-sealed tunnel, a complementary ensemble empirical mode decomposition with adaptive noise and multiscale permutation entropy and Hilbert–Huang transform (HHT) method was proposed. Then, the proposed method was verified by the numerical simulation and the Huangdao groundwater-sealed tunnel engineering. The results show that the proposed method can suppress modal aliasing and signal noise and identify the blasting characteristics of groundwater-sealed tunnel effectively. In addition, the blasting vibration energy which accounts for 94.7% in the frequency range of 0–200 Hz, 72.5% of 0–50 Hz was summarized. Furthermore, the safety status of each monitoring point was evaluated through HHT and the feasibility of millisecond blasting was identified. The method proposed can identify the vibration characteristics and safety status of groundwater-sealed tunnel from the perspective of time–frequency and energy effectively.

## Introduction

Blasting, which is an economical and effective means of excavation, is widely used in mining, railway, highway tunnel, hydropower engineering, groundwater-sealed tunnel, and demolition of urban high-rise buildings^1–3^. Groundwater-sealed tunnel storage refers to an underground space system that utilizes the principle of water sealing to store oil and gas energy, excavated in a certain depth of rock below a stable groundwater level. It is known as a “highly strategic and safe reserve reservoir” by the global industry and has become the main storage method for energy such as oil and liquefied gas internationally. Groundwater-sealed tunnel is in a dynamic underground water environment, with many adjacent caverns, high anti-seepage requirements, strict blasting control, etc., making tunnel stability and safety control the basis for construction and safe operation. During its blasting and excavation, a portion of the blast energy is used to break the rock mass (deform, destroy, move, and throw the rock mass, etc.) while performing work on the rock mass around the blast hole. Meanwhile, another portion of the energy will be dynamically propagated to the rock mass in the form of blasting seismic waves, causing vibration and damage effects on adjacent tunnels and supporting facilities^4,5^. The propagation law, waveform characteristics, energy characteristics and attenuation law of blasting seismic waves in the medium can be revealed by monitoring, extracting, and analysing the information in the blasting vibration signal and studying the frequency spectrum and energy distribution characteristics of the blasting vibration signal^6–8^. The analysis and evaluation of blasting vibration is of great significance to the quality effect of blasting construction and the safety and stability of adjacent tunnels^9^. However, the traditional feature identification methods are mainly applied to common tunnels, mines, and slopes excavation, which are less commonly researched in groundwater-sealed tunnel. Therefore, it is necessary to study the identification of blasting characteristics for groundwater-sealed tunnel.

The overall blasting vibration signal presents non-stationary and nonlinear characteristics due to the influence of certain factors, such as complex environment, electromagnetic interference, and monitoring instrument errors^10^. Some scholars use certain current time–frequency analysis techniques to identify and analyse the signal. Fast Fourier transform (FFT) converts the signal from the entire time domain to the frequency domain, analyses the dynamic change and attenuation characteristics of the blasting seismic wave energy with time and instantaneous frequency and distinguishes different types of waveforms^11^. Wavelet analysis can realise time–frequency analysis, multi-band analysis and energy distribution characteristic analysis and identify the energy distribution characteristics of blasting seismic waves with multiple frequency bands compared with previous methods, which can only be analysed from a single element, such as the amplitude, frequency and duration of blasting vibration waves^12^. The empirical mode decomposition (EMD) proposed by Huang et al.^13^ can perform multi-layer adaptive decomposition for the characteristics of non-stationary and nonlinear signals and obtain the intrinsic mode function (IMF)^14^, which contains different characteristic time scales and has its own physical meaning that can highlight the local features of the signal and perform multi-resolution analysis^15^. The ensemble EMD (EEMD) and complementary EEMD (CEEMD) methods are improved based on EMD by adding Gaussian white noise. These methods divide the original signal into components of different scales in the time–frequency space, are all adaptive, noise-assisted data analysis methods^16^, can solve the problem of modal aliasing to a certain extent and realise adaptive decomposition and time–frequency feature extraction of non-stationary signals^17^. The CEEMD with adaptive noise (CEEMDAN), which is also improved on the basis of EMD by adaptively adding white noise, reduces the phenomenon of modal aliasing, overcomes the problem of reconstruction error and can accurately reconstruct the original signal^18^.

Although the Fourier transform can describe the entire frequency distribution, it cannot simultaneously reflect the overall or local characteristics of the signal in the time and frequency domains. The application conditions are harsh, and certain problems, such as frequency component confusion for non-stationary signals, occur. The wavelet transform (WT) is a type of Fourier transform with an adjustable window, but it cannot avoid the limitations of Fourier transform. In addition, the selection of the wavelet base will affect the results, and the limited length of the wavelet will cause leakage of signal energy, making it difficult to quantitatively analyse the signal energy–frequency–time distribution characteristics^19^. EMD often contains severe modal aliasing and end-point divergence effects when decomposing noisy blasting seismic waves to obtain IMF, which will affect the accuracy of the signal analysis to a certain extent^20^. Although EEMD and CEEMD can divide the original signal into different scale components in the time–frequency space by adding Gaussian white noise, they cannot completely remove the added white noise during reconstruction and avoid the influence of residual noise, resulting in distortion of the original signal^21^. CEEMDAN can solve the modal aliasing phenomenon caused by the traditional decomposition method, obtain pure main modal components and improve the adaptability of subsequent HHT analysis^22^. However, CEEMDAN still cannot avoid the influence of residual noise^23^. Meanwhile, the time–frequency energy analysis method based on EMD or HHT is mainly used to identify and analyse the blasting vibration characteristics of conventional tunnel and mining engineering, rarely involved in groundwater-sealed tunnel engineering. The identification and analysis of blasting vibration characteristics of groundwater-sealed tunnel will have far-reaching significance for the damage effect of rock mass vibration and the safety control of adjacent tunnels.

To solve the above-mentioned problems in blasting vibration signal processing, analyse and evaluate the blasting vibration characteristics, safety and stability in groundwater-sealed tunnel, this work employs multi-scale permutation entropy (MPE) as a detection method of signal randomness and dynamic mutation to detect the complexity and randomness of IMFs derived from CEEMDAN decomposition, eliminating noise or spurious components to achieve signal noise reduction and purification, and provide feasibility for the subsequent Hilbert transform^24^. The CEEMDAN-MPE method has much advantages in terms of denoising and adaptive discrimination compared to other methods. Meanwhile, in feature extraction and analysis, Hilbert–Huang transform (HHT), a new method of processing nonlinear and non-stationary signals, breaks the limitation of Heisenberg’s uncertainty principle and accurately reflects the change characteristics of signals in the time–frequency domain and energy. Previous research on blasting vibration signals mainly considers single factors, such as frequency or amplitude, through FFT, WT, etc. In this work, the HHT analysis method is employed to reflect the specific distribution of vibration signal energy comprehensively and clearly with time and frequency and identity the blasting vibration signal characteristics from the perspective of time–frequency and energy effectively.

In view of the problem that the traditional feature identification methods are less explored to groundwater-sealed tunnel, this work proposes an abnormal signal identification and noise reduction method of CEEMDAN-MPE, which conducts signal decomposition and noise reduction and constructs an analogue simulation signal to compare and verify the proposed method with EMD, WT and other related methods. Then, the above-mentioned methods in combination with the HHT time–frequency and energy characteristic analysis method is applied to the Huangdao groundwater-sealed tunnel engineering. First, the time–frequency and energy features of the blasting vibration signals are extracted and analysed. Moreover, the time–frequency and energy distribution of the signal, the attenuation law of energy propagation, the safety status and the identification of the blasting delay of the groundwater-sealed tunnel are discussed. It is shown that the method proposed in this work can help in identifying the characteristics of blasting vibration signals and evaluating the safety of groundwater-sealed tunnel.

## Feature identification methods

### CEEMDAN decomposition principle

The CEEMDAN^25^ method decomposes the signal by adding adaptive white noise and calculating the only residual component to eliminate the modal aliasing phenomenon existing in EMD. This method also solves the transfer problem of white noise from high frequency to low frequency and achieves adaptive time–frequency decomposition of non-stationary signals and a certain degree of noise reduction. The realisation principle is as follows:

*Step* 1: Original signal: $X\left( t \right)$

$v^{i} \left( t \right)$ is a Gaussian white noise signal with standard normal distribution, and $\varepsilon$ is the standard deviation of the noise, adding positive and negative pairs of Gaussian white noise to the original signal for the experiment. The $i$ time experiment can be expressed as a new signal:

1 $$X^{i} \left( t \right) = X\left( t \right) + \varepsilon v^{i} \left( t \right)$$

where $$i = 1, 2, 3, \ldots , I$$.

*Step* 2: $X^{i} \left( t \right)$ is decomposed by EMD to obtain $IMF_{1}$. Then, the summed average of the components is calculated to obtain the first-order modal components:

2 $$IMF_{1} = \frac{1}{I}\mathop \sum \limits_{i = 1}^{I} IMF_{1}^{i}$$

Residuals of the first-order modal components:

3 $$r_{1} \left( t \right) = X\left( t \right) - IMF_{1}$$

*Step* 3: A positive and negative paired Gaussian white noise $\varepsilon v^{i} \left( t \right)$ is added to residual $r_{1} \left( t \right)$, and $i$ time experiments are performed to obtain:

4 $$X^{i} \left( t \right) = r_{1} \left( t \right) + \varepsilon v^{i} \left( t \right)$$

EMD is performed to obtain $IMF_{2}$. Then, the summed average of the components is calculated to determine the second-order modal component:

5 $$IMF_{2} = \frac{1}{I}\mathop \sum \limits_{i = 1}^{I} IMF_{2}^{i}$$

Residuals of the second-order modal components:

6 $$r_{2} \left( t \right) = r_{1} \left( t \right) - IMF_{2}$$

*Step* 4: Steps 2 and 3 are repeated until the residual component is a monotonic function, and the decomposition cannot be continued. Then, the algorithm ends. The number of eigenmodes currently is $K$. Subsequently, signal $X\left( t \right)$ can be decomposed into:

7 $$X\left( t \right) = \mathop \sum \limits_{k = 1}^{K} IMF_{k} + r_{K} \left( t \right)$$

### MPE detection method

CEEMDAN can realise adaptive time–frequency decomposition of non-stationary signals and noise reduction to a certain extent. However, this algorithm does not isolate residual noise. Accordingly, the added white noise can always be transferred from high frequency to low frequency, resulting in IMF residual white noise signal. The MPE^26^ is a method for detecting the randomness and dynamic mutation of the signal. The complexity and randomness of each component decomposed can be detected by using MPE, and the noise component can be eliminated to achieve signal noise reduction. MPE performs multi-scale coarse-graining of the time series and calculates its permutation entropy. The implementation principle is as follows:

*Step* 1: Coarse-grained processing on sequence $X = \left\{ {x\left( i \right),i = 1,2,3,...,n} \right\}$ is performed to obtain the processed coarse-grained sequence:

8 $$y^{s} \left( j \right) = \frac{1}{s}\mathop \sum \limits_{{i = \left( {j - 1} \right){*}s + 1}}^{{j{*}s}} x\left( i \right)$$

where $s$ is the scale factor, and $y^{s} \left( j \right)$ is the time series under different scale factors.

*Step* 2: Time series $y^{s} \left( j \right)$ is reconstructed to obtain:

9 $$Y_{t}^{s} = \left\{ {y_{t}^{s} ,y_{t + \tau }^{s} ,...,y_{{t + \left( {m - 1} \right){*}\tau }}^{s} } \right\}$$

where $m$ is the embedding dimension, and $t$ is the delay time.

*Step* 3: The permutation entropy of the time series under scale factor $s$ is calculated:

10 $$H_{p}^{s} \left( m \right) = - \mathop \sum \limits_{j = 1}^{m!} P_{j}^{s} {*}\ln P_{j}^{s}$$

where $P_{j}$ is the probability of the time occurrence of the symbol sequence.

*Step* 4: The permutation entropy calculated above is normalised:

11 $$h_{P}^{s} = H_{p}^{s} /\ln (m!)$$

### HHT feature analysis method

HHT^13^ is a new method for dealing with nonlinear and unstable signals. This method generates sequences with distinct characteristics by decomposing signals at various scales, which can accurately reflect the distribution law of signals on different spaces or time scales. HHT is composed of two parts: EMD and Hilbert transform. The signal is decomposed into different IMFs through EMD to provide actual physical meaning for the instantaneous frequency. Meanwhile, Hilbert transform is performed on different IMFs to obtain the Hilbert spectrum and comprehensively describe the time–frequency and energy spectrum of the signal. The Hilbert transform is as follows:

For time series: $F\left( t \right)$, the Hilbert transform is as follows:

12 $$G\left( t \right) = \frac{1}{\pi }K\mathop \smallint \limits_{ - \infty }^{\infty } \frac{F\left( \delta \right)}{{t - \delta }}d\delta$$

where $K$ is the principal value of Cauchy, when $F\left( t \right)$ and $G\left( t \right)$ are complex conjugates, and an analytical signal $P\left( t \right)$ can be obtained:

13 $$P\left( t \right) = F\left( t \right) + jG\left( t \right) = a\left( t \right)e^{j\alpha \left( t \right)}$$

where instantaneous amplitude: $a\left( t \right) = \sqrt {F^{2} \left( t \right) + G^{2} \left( t \right)}$; instantaneous phase: $\alpha \left( t \right) = \arctan \frac{G\left( t \right)}{{F\left( t \right)}}$; and instantaneous angular frequency: $\omega = \frac{d\alpha \left( t \right)}{{dt}}$.

The Hilbert transform provides a unique function for finding the instantaneous frequency and instantaneous amplitude. Hilbert transform is performed on each IMF component to obtain the Hilbert spectrum:

14 $$H\left( {\omega ,t} \right) = {\text{Re}} \left[ {\mathop \sum \limits_{i = 1}^{n} a_{i} \left( t \right)\exp (j\smallint \omega_{i} \left( t \right)dt)} \right]$$

(a) The distribution of amplitude in frequency and time can be obtained from the above formula. Then, the Hilbert spectrum is integrated with time to obtain the marginal spectrum:

15 $$h\left( \omega \right) = \mathop \smallint \limits_{ - \infty }^{\infty } H\left( {\omega ,t} \right)dt$$

The Hilbert marginal spectrum represents the accumulation of the amplitude or energy at each frequency in time and can reflect the energy concentration at each frequency.

(b) The square of the amplitude over time is integrated to determine the energy spectrum:

16 $$ES\left( \omega \right) = \mathop \smallint \limits_{ - \infty }^{\infty } H^{2} \left( {\omega ,t} \right)dt$$

The Hilbert energy spectrum reflects the energy accumulation of each frequency over the entire length of time.

(c) The square of the amplitude against the frequency is integrated to obtain the instantaneous energy spectrum:

17 $$IE\left( t \right) = \mathop \smallint \limits_{\omega } H^{2} (\omega ,t)d\omega$$

The Hilbert instantaneous energy spectrum reflects the accumulation and variation of the waveform energy in the entire frequency domain at each moment.

## Simulation analysis

**Figure 1:**
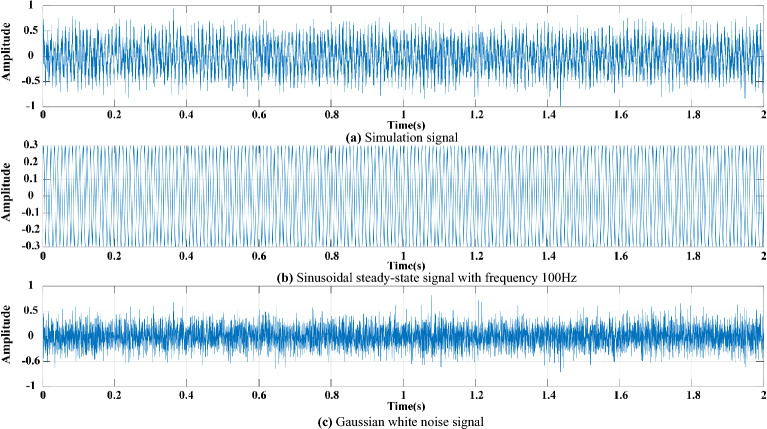
(**a**) Simulation signal. (**b**) Sinusoidal signal. (**c**) Noise signal.

An analogue simulation signal is constructed to verify the feasibility of CEEMDAN-MPE noise reduction filtering and feature identification proposed in this work. The signal is composed of a stationary sine function with a frequency of f = 100 Hz and a Gaussian white noise with RMSE = 0.2. The sampling frequency is 4 kHz, the sampling time is t = 2 s, and the number of sampling points is N = 8000. The synthetic signal, sine signal and noise are obtained as shown in Fig. 1.

**Figure 2:**
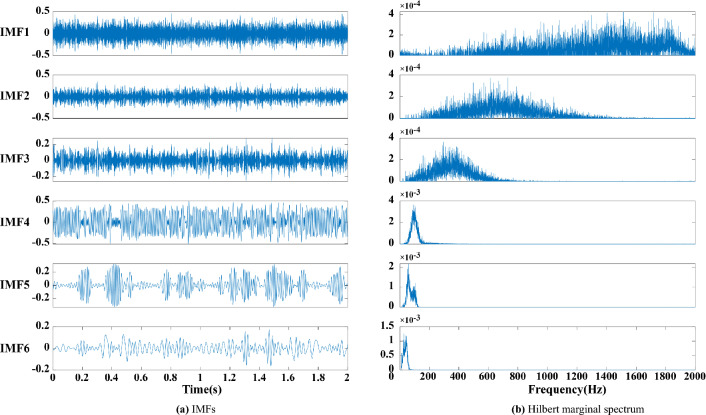
(**a**) EMD decomposition. (**b**) Hilbert marginal spectrum.

Four methods, namely, EMD, EEMD, CEEMDAN and CEEMDAN-MPE, are used for the signal processing of the above analogue signals to obtain the first six decomposed IMF components and the Hilbert marginal spectrum of each component, as shown in Fig. 2.

After denoising the simulated signal by CEEMDAN, the MPE of each IMF is calculated, as shown in Table 1, and the randomness and complexity of each IMF component are evaluated. After many trial calculations and references^27^, we determined that this component can be considered an abnormal component with considerable randomness when MPE > 0.6 and must be eliminated. After removing abnormal components, a new signal is obtained by reconstruction. Then, CEEMDAN is performed to obtain the final IMF and Hilbert marginal spectrum, as shown in Fig. 5, and the signals before and after processing are compared in Fig. 6.

**Table 1:** MPE of each IMF component after CEEMDAN decomposition.

Component	IMF1	IMF2	IMF3	IMF4	IMF5	IMF6	IMF7	IMF8	IMF9	IMF10
MPE	0.91	0.86	0.76	0.59	0.45	0.34	0.18	0.20	0.16	0.13

Component	IMF11	IMF12	IMF13	IMF14	IMF15					
MPE	0.12	0.114	0.110	0.104	0.100					

In addition, in order to verify the processing ability of the CEEMDAN-MPE method for the simulation signal, the denoising effects of Wavelet and Wavelet Package were compared, and the results were obtained according to the SNR (signal to interference plus noise ratio) and RMSE (root mean squared error) indicators as shown in Table 2. It can be seen that the proposed method has the highest SNR and the lowest RMSE, which can better extract the true signal components from the simulation signal and provide a foundation for subsequent feature extraction.

**Table 2:** Comparison of simulation signal processing effects.

Processed methods	SNR	RMSE
CEEMDAN + MPE	16.856	0.1841
Wavelet	15.042	0.1966
Wavelet package	7.077	0.2146

**Figure 3:**
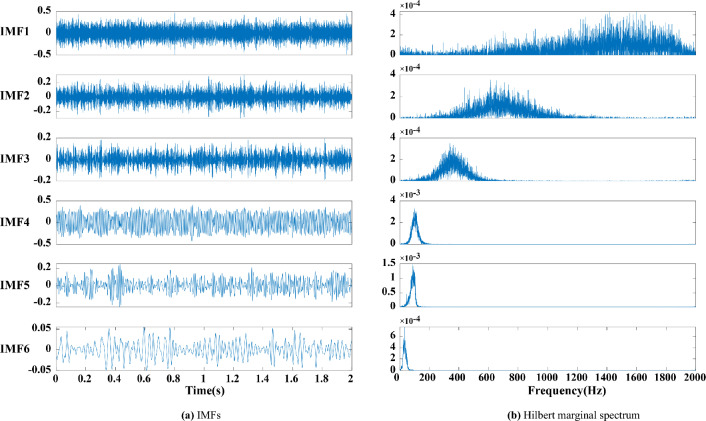
(**a**) EEMD decomposition. (**b**) Hilbert marginal spectrum.

**Figure 4:**
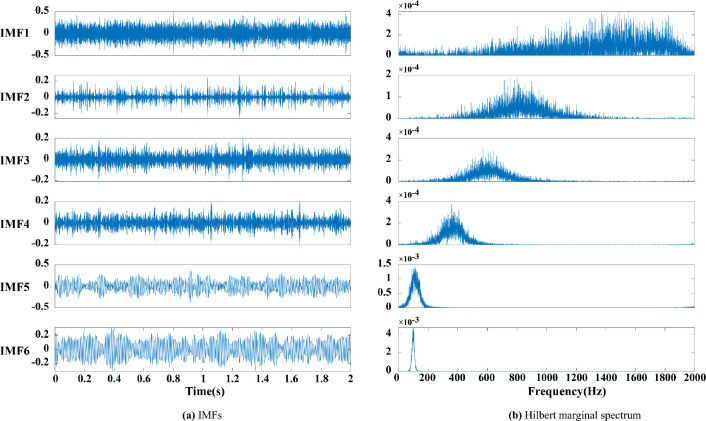
(**a**) CEEMDAN decomposition. (**b**) Hilbert marginal spectrum.

**Figure 5:**
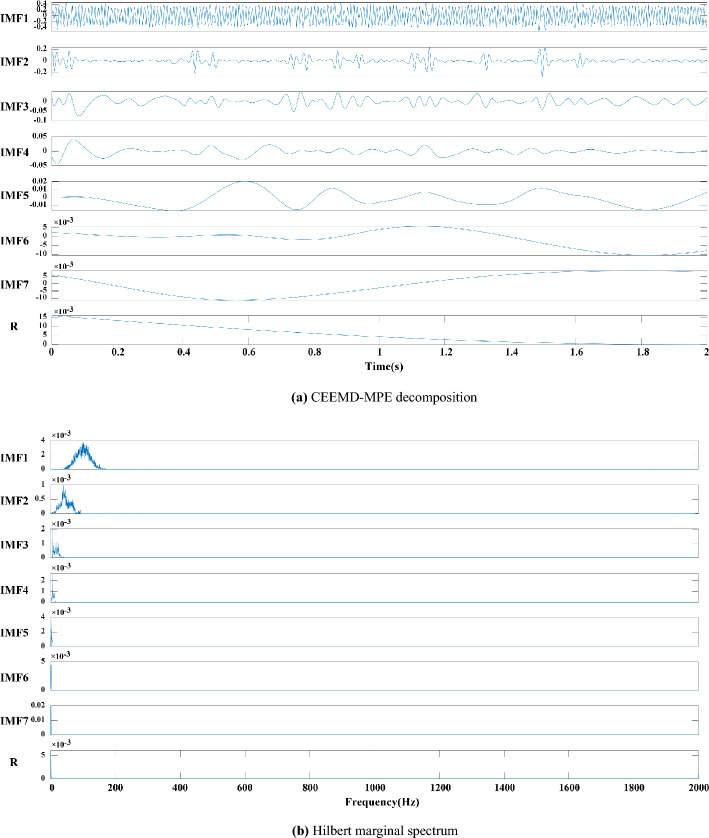
(**a**) CEEMDAN-MPE decomposition. (**b**) Hilbert marginal spectrum.

**Figure 6:**
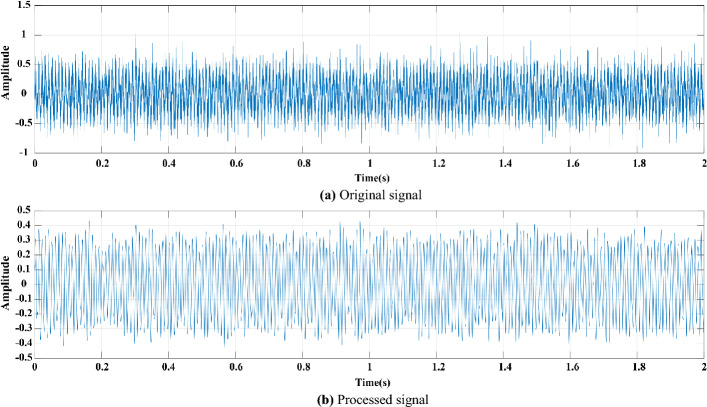
Comparison before and after CEEMDAN-MPE. (**a**) Original signal. (**b**) Processed signal.

The following conclusions can be drawn by analysing Figs. 2, 3, 4, 5 and 6:

(1) The high frequency components decomposed by EMD have an obvious modal aliasing phenomenon. EEMD and CEEMDAN have false components under the interference of noise (i.e., components greater than 250 Hz), and the false components are difficult to identify. The modal aliasing occurring in high frequencies still exists in the component.

(2) The first four components of EMD, the first three components of EEMD and the first four components of CEEMDAN have an obvious modal aliasing phenomenon. According to the analysis of the Hilbert marginal spectrum corresponding to each component, the frequencies of the first three components of EMD, EEMD and CEEMDAN exceed 250 Hz, far exceeding the 100 Hz frequency of the actual signal. This finding indicates that this component contains considerably high-frequency noise, but its low-frequency component is relatively stable.

(3) The modal aliasing phenomenon still exists in the simple use of CEEMDAN, and the first three IMFs have high-frequency noise components. MPE is adopted to detect the randomness and complexity of each component. After removing the abnormal components, the remaining signals are reconstructed and decomposed to obtain seven IMFs and a trend item R as shown in Fig. 5, where the modal aliasing phenomenon of the decomposed IMFs is suppressed. Based on the Hilbert marginal spectrum analysis of each component, the frequency of each component is within the 100 Hz signal of the simulated signal, and the signal energy is mainly concentrated in the first five components.

(4) The result of the comparison of the original signal and the signal processed by CEEMDAN-MPE (Fig. 6) demonstrates that the decomposition and noise reduction method proposed in this work can effectively reduce the burrs and irregularities in the signal whilst retaining the original signal feature information and better reflect the signal waveform characteristics.

In summary, the CEEMDAN-MPE method designed in this work can eliminate the modal aliasing phenomenon of the original signal, effectively detect abnormal components, remove high-frequency noise, retain real information and improve the analysis accuracy compared with the traditional method. This method can provide a scientific and effective approach for the identification and analysis of the characteristics of the subsequent blasting vibration signals.

## Engineering application analysis

### Project overview and on-site monitoring

The Huangdao groundwater-sealed tunnel blasting engineering is located in Huangdao District, Qingdao City. This project is the first large-scale groundwater-sealed tunnel oil depot project in China. The designed storage capacity is 3 million m3, which is divided into two individual projects: underground and above-ground. The underground project has a total of three groups of tunnels, consisting of nine main tunnels, five water curtain tunnels, six process shafts, construction tunnels, ventilation tunnels, etc. The section of the main tunnel is a straight wall and circular arch, with a span of 20 m, a height of 30 m and a total length of 5.6 km. The rock mass type is mainly grade II.

**Figure 7:**
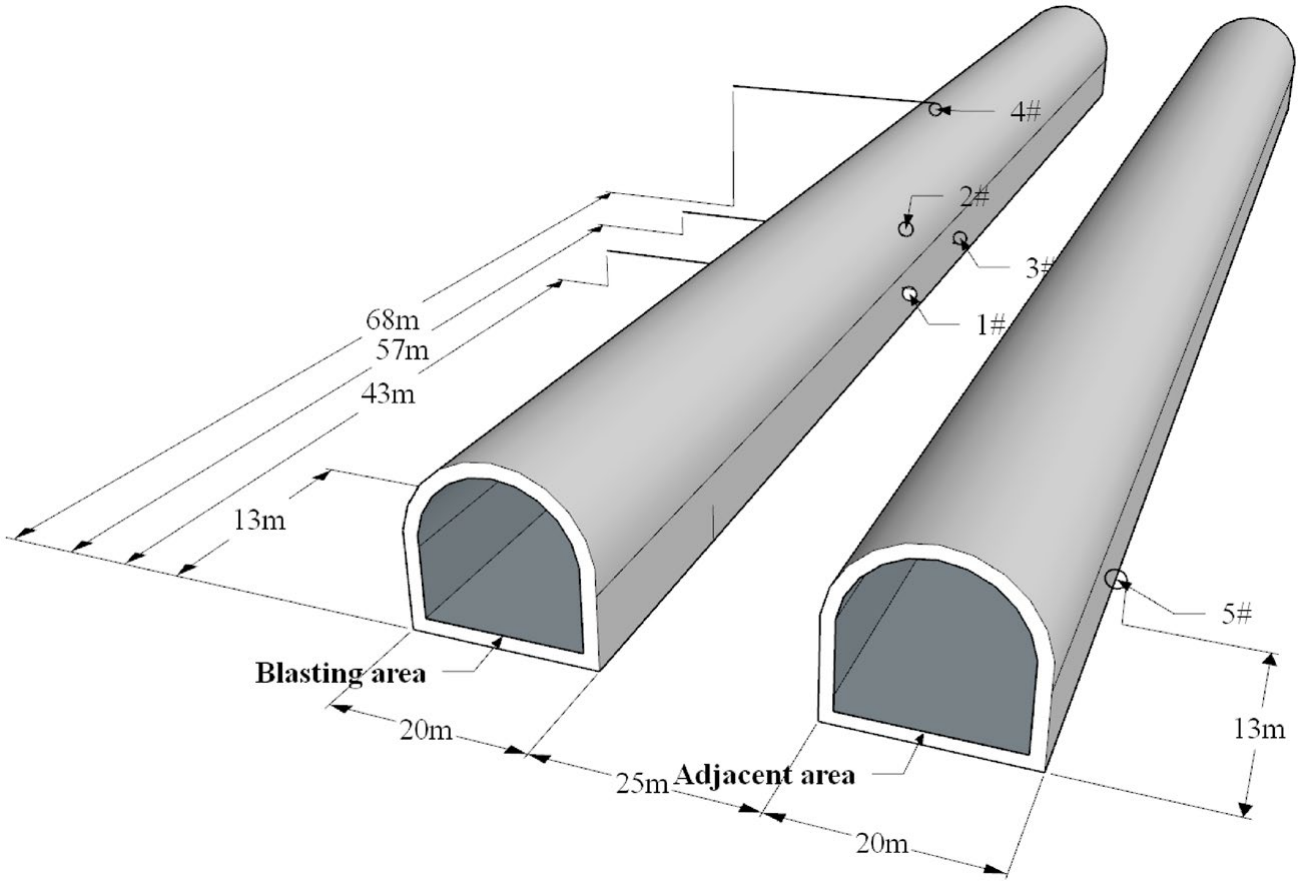
The layout of blasting vibration measuring point.

**Figure 8:**
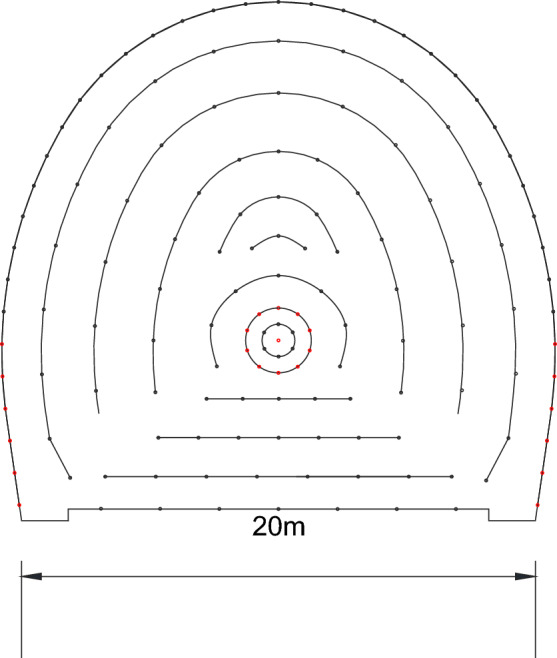
The layout of blasting section.

**Figure 9:**
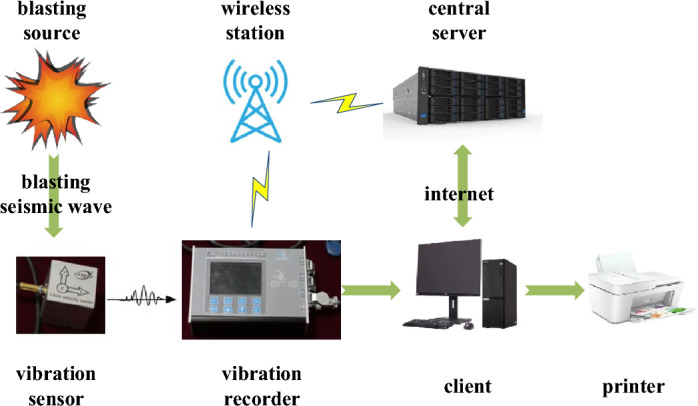
The blasting vibration meter and monitoring system.

**Figure 10:**
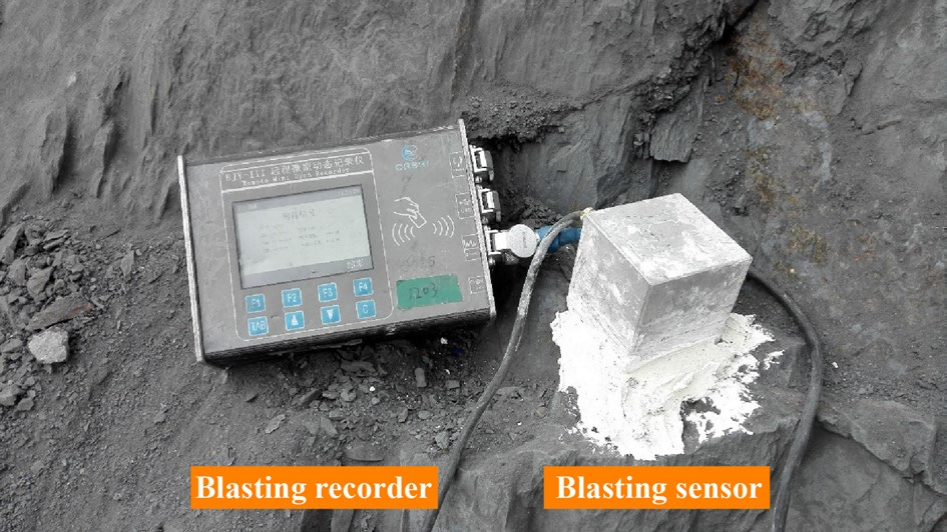
The monitoring of blasting vibration on the groundwater-sealed tunnel.

The layout of the site blasting vibration monitoring measuring points is shown in Fig. 7. At the blasting area rear, the 1# and 3# measuring points are arranged on the rock wall at the foot of the side wall; the 2# measuring point is arranged on the side wall bolt; the 4# measuring point is arranged on the top of the main tunnel; and the 5# measuring point is arranged in the adjacent tunnel. The layout of blasting section is shown in Fig. 8. The independently developed blasting recorder BJY-III is used for monitoring. The monitoring system shown in Fig. 9 is established, the sampling frequency is f = 5 kHz, and each measuring point collects the particle vibration velocity in three directions. The on-site blasting vibration monitoring is shown in Fig. 10. The blasting design parameters, detonator initiation stage and section charge are shown in Tables 3 and 4.

**Table 3:** The blasting design parameter.

Blasting method	Hole spacing (m)	Row spacing (m)	Hole diameter (mm)	Hole depth (m)	Specific consumption (kg m^−3^)	Explosive type	Delay time (ms)
Bench blasting	3	2.5	90	11.5	0.5	Emulsion explosive	50

**Table 4:** The detonator section quantity and section charge quantity.

Section	1	3	5	7	15	Total
Detonator quantity	1	28	8	6	113	156
Explosive quantity (kg)	10.8	287.8	72.5	60.0	800.9	1232

**Figure 11:**
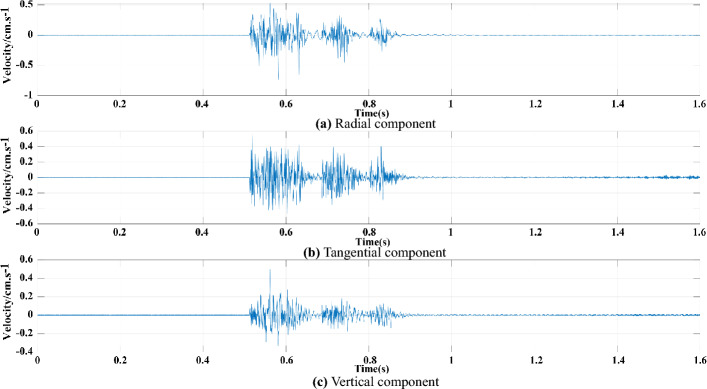
Blasting vibration velocity time-series. (**a**) Radial component. (**b**) Tangential component. (**c**) Vertical component.

In combination with the on-site blasting vibration monitoring scheme, a typical measuring point so-called 4# measuring point is selected as an example for the signal extraction and analysis. The original signals of radial, tangential and vertical vibration monitoring are shown in Fig. 11.

### Analysis of filter noise reduction

**Figure 12:**
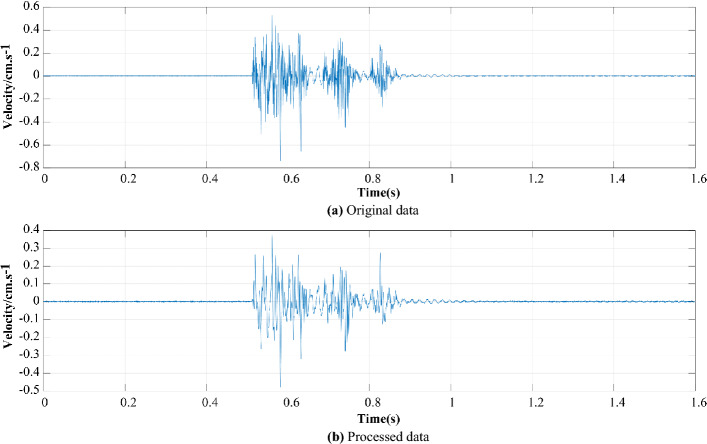
Comparison between the original data and the processed data. (**a**) Original data. (**b**) Processed data.

The CEEMDAN-MPE method proposed in this work is adopted to process the signal in the radial direction of the 4# measuring point, as shown in Fig. 12. In Fig. 12, the overall shape of the signal after filtering and noise reduction is intact, and the noise is removed.

**Figure 13:**
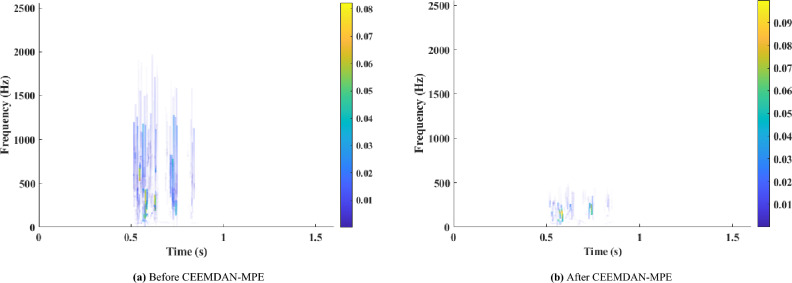
The Hilbert spectrum. (**a**) Before CEEMDAN-MPE. (**b**) After CEEMDAN-MPE.

The Hilbert spectrum before and after blasting vibration signal processing is compared, as shown in Fig. 13. The frequency of the original signal is between 0 and 2000 Hz, and that of the processed signal is between 0 and 500 Hz. The noise signals higher than 500 Hz have been effectively removed, which is consistent with the general distribution law of tunnel blasting vibration frequency^28^.

### Hilbert marginal spectral distribution characteristics

**Figure 14:**
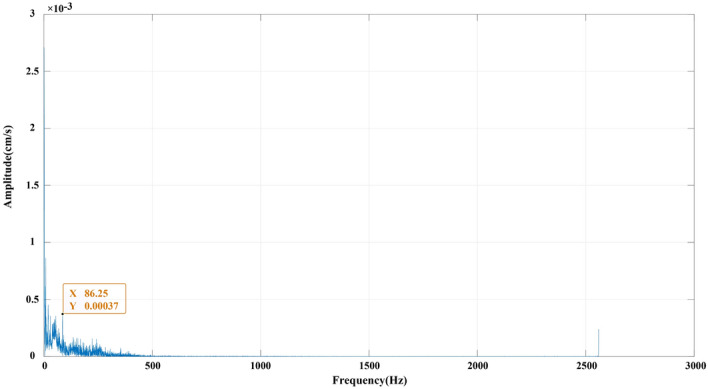
The Hilbert marginal spectrum of 4# measuring point in the radial component.

**Figure 15:**
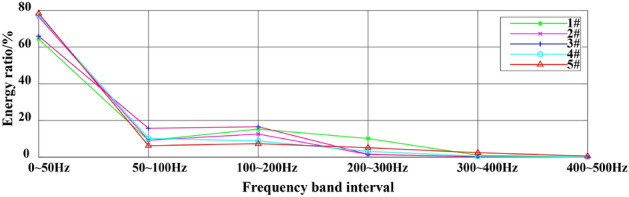
The energy ratio (%) of each frequency band at every measuring point.

The Hilbert marginal spectrum is obtained from the radial component of the 4# typical measuring point through the method proposed in this work, and the amplitude distribution of different frequency bands is shown in Fig. 14. Meanwhile, the vibration frequency is divided into six frequency bands to quantitatively describe the energy carried by each frequency band: 0–50 Hz, 50–100 Hz, 100–200 Hz, 200–300 Hz, 300–400 Hz and 400–500 Hz. The squares of the radial, tangential and vertical Hilbert spectra of the 1#–5# measurement points are integrated in each frequency band interval, and the energy ratio of each frequency band interval is counted to obtain the data shown in Table 5. The cumulative average energy proportion of the first three frequency bands (0–200 Hz) are counted, and the energy proportion of each frequency band at every measuring point is obtained, as shown in Fig. 15.

**Table 5:** The energy ratio (%) of each frequency band at every measuring point.

Measuring point	Component	Frequency band interval						Accumulation of the first three frequency bands
		0–50 Hz	50–100 Hz	100–200 Hz	200–300 Hz	300–400 Hz	400–500 Hz	
1#	Radial	0.523143	0.071736	0.243252	0.141424	0.019346	0.001099	
	Tangential	0.56236	0.134961	0.151142	0.144917	0.006438	0.000183	
	Vertical	0.845473	0.065851	0.064	0.018063	0.005255	0.001357	
	Avg.	0.643659	0.090849	0.152798	0.101468	0.010346	0.000879	0.887306
2#	Radial	0.776731	0.106177	0.101332	0.013067	0.002381	0.000312	
	Tangential	0.68231	0.100884	0.198598	0.016459	0.001517	0.000232	
	Vertical	0.834695	0.06897	0.077705	0.014655	0.003409	0.000568	
	Avg.	0.764579	0.09201	0.125878	0.014727	0.002435	0.000371	0.982467
3#	Radial	0.652709	0.176382	0.148541	0.019953	0.002195	0.00022	
	Tangential	0.569219	0.145259	0.269545	0.014044	0.001612	0.000322	
	Vertical	0.756772	0.149366	0.079028	0.012223	0.002373	0.000239	
	Avg.	0.659566	0.157002	0.165705	0.015407	0.00206	0.00026	0.982273
4#	Radial	0.6912	0.198866	0.068049	0.035903	0.004839	0.001143	
	Tangential	0.740422	0.066189	0.142694	0.043752	0.006398	0.000545	
	Vertical	0.887803	0.041264	0.052896	0.015267	0.00243	0.00034	
	Avg.	0.773142	0.102106	0.08788	0.031641	0.004556	0.000676	0.963128
5#	Radial	0.909833	0.040543	0.028099	0.009591	0.007056	0.004879	
	Tangential	0.658081	0.075531	0.10293	0.105957	0.050463	0.007038	
	Vertical	0.782628	0.070969	0.088323	0.038099	0.014851	0.00513	
	Avg.	0.783514	0.062348	0.073117	0.051216	0.024123	0.005682	0.918979
Accumulation Avg.		0.724892						0.946831

(1) Fig. 13 shows that the blasting vibration signal energy is mainly concentrated in the low-frequency region within 200 Hz. The cumulative amplitude is relatively large in the frequency range less than 86 Hz. This finding demonstrates that the blasting vibration frequency will develop towards the low frequency direction (< 86 Hz), the amplitude quickly drops after 86 Hz, and the amplitude will progressively attenuate and tend to zero after 200 Hz.

(2) Table 5 and Fig. 14 demonstrate that the frequency within 0–50 Hz carries the most average energy, accounting for 72.5% of the total energy, indicating that it is the main frequency band. The frequency within 200 Hz occupies majority of the signal’s energy, accounting for 94.7%, indicating that the vibration frequency of blasting vibration is within 200 Hz.

(3) When the average energy proportions of the radial, tangential and vertical components of the 1#–5# measuring points in Table 5 are compared, the vertical component energy proportion is the largest in the main frequency band (0–50 Hz). This result indicates that the vibration load caused by the vertical direction is the largest, and the vertical direction of the tunnel rock mass is most likely to be damaged. However, the 5# measuring point in the adjacent tunnel has the largest proportion of radial component energy, and its radial direction has the greatest impact.

### Hilbert instantaneous energy distribution characteristics

**Figure 16:**
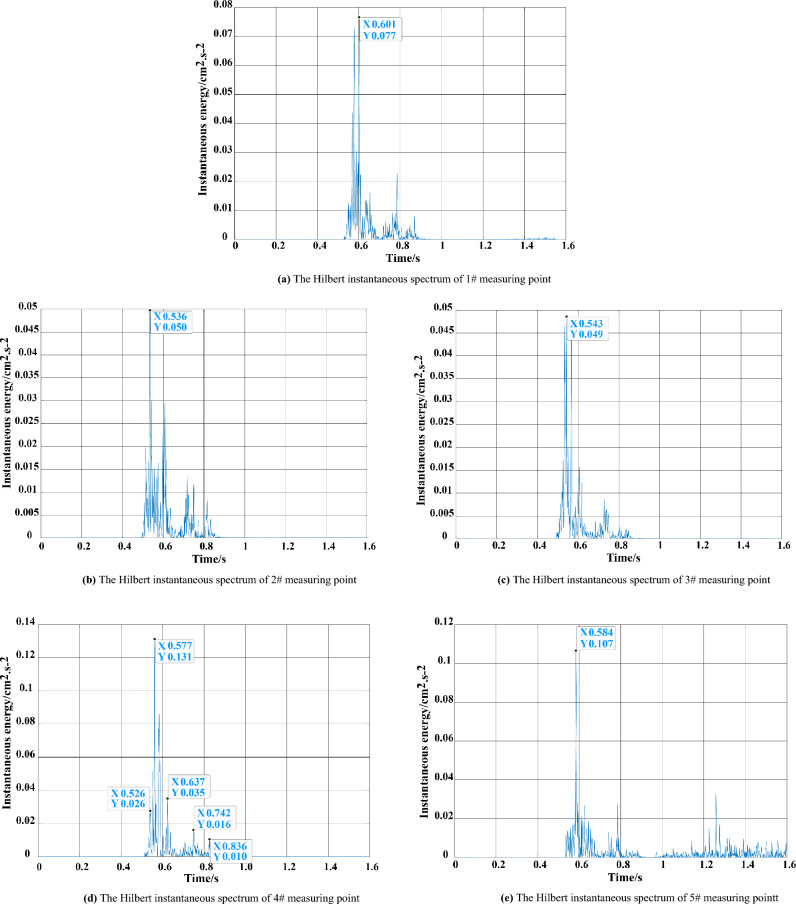
The Hilbert instantaneous spectrum. (**a**) 1# measuring point. (**b**) 2# measuring point. (**c**) 3# measuring point. (**d**) 4# measuring point. (**e**) 5# measuring point.

The square of the Hilbert spectrum in the radial component of the 1#–5# measurement points is integrated over the entire frequency range through the method proposed in this work. The Hilbert instantaneous energy (cm^2^ s^−2^) spectrum of each measurement point in the radial component can be obtained, as shown in Fig. 16.

Accurate identification of the delay time in differential blasting is of great significance for blasting design optimisation and reducing blasting vibration effects^29^. Taking the vertical direction of the 4# measuring point as an example, the Hilbert instantaneous energy spectrum obtained is shown in Fig. 16d. Each peak of the instantaneous energy spectrum represents the energy release caused by the detonator explosion. The five peaks represent the 1st, 3rd, 5th, 7th and 15th detonator detonating time. Meanwhile, the theoretical delay time, actual delay time and interval of each section of the detonator are counted, as shown in Table 6.

**Table 6:** Comparison of the detonators delay time.

Section	Theoretical delay time (ms)	Theoretical delay interval (ms)	Actual delay interval (ms)	Actual delay time (ms)
1	< 13	–	–	0
3	50 ± 10	27–60	51	51
5	110 ± 15	35–85	60	111
7	200 ± 20	55–125	105	216
15	880 ± 60	600–760	94	310

According to Fig. 16 and Table 6:

(1) The instantaneous energy of blasting vibration increases first and then decreases, and several sub-peaks appear after the peak. Taking the vertical component of the 4# measuring point as an example, the instantaneous energy of the blasting vibration signal lasts from 0.51 to 0.86 s after detonation. The duration is 0.35 s and reaches the peak at 0.58 s. Thereafter, three more sub-peaks become evident, and the interval of which is about within 100 ms. The signal energy shows a gradual decay trend and conforms to the law of the differential blasting time interval of 50–110 ms^20^.

(2) The instantaneous energy peak represents the maximum load of blasting vibration, which causes a large structural displacement increment^30^. If the instantaneous energy is considerably large, then the stability of the rock mass can be easily affected. The instantaneous energy peaks of the 1#, 2# and 3# measuring points (from near to far from the blasting area) located at the foot of the sidewall and on the sidewall bolt are 0.077, 0.05 and 0.049 cm^2^ s^−2^, respectively, which are smaller than 0.131 cm^2^ s^−2^ of the 4# (at the top arch of the tunnel) and 0.107 cm^2^ s^−2^ of the 5# (in the adjacent tunnel) measuring points. We can preliminarily conclude that the blasting vibration has little influence on the safety of the sidewall as well as the sidewall bolt support, and the effect of the bolt support is obvious. In addition, the 5# measuring point has no obvious energy fluctuation after reaching the energy peak at 0.58 s, and its vibration attenuation amplitude is the largest compared with other measuring points. This notion indicates that the blasting vibration has less impact on the adjacent tunnel, which can guarantee the stability of the rock mass in the adjacent tunnel during blasting excavation.

(3) The instantaneous energy of the 4# measuring point located on the top arch of the tunnel is the largest (0.131 cm^2^ s^−2^), which is greater than those of all other measuring points. This notion indicates that the impact of blasting vibration on the top arch of the tunnel is greater than that of the sidewall of the tunnel. It’s best to be strengthened with roof arch monitoring and support.

(4) In the whole vibration time course, the 2nd peak is the largest, the 3rd peak is smaller than the 2nd, and the 4th peak is smaller than the 3rd peak, which is consistent with the design charge of the 3rd section with the largest charge and the 7th section with a smaller charge than the 5th section. Meanwhile, Table 6 illustrates that the actual delay intervals of the first four sections of detonators are basically within the range of the theoretical delay intervals. The calculated actual detonation time of each detonator is also within the theoretical detonation time, and the overall blasting effect is good. However, the amount of charge in the 15th section is considerably large, the energy peak is small and premature explosion occurs, indicating the charge structure of the 15th section detonator should be further improved (Supplementary material).

## Conclusion

In view of the problem that the traditional feature identification methods are less explored to groundwater-sealed tunnel, the CEEMDAN-MPE-HHT method was proposed in this work. The method aims at its characteristics of blasting excavation and safety protection requirements, combining the adaptability of signal decomposition and the ability to detect signal anomalies, and can identify vibration characteristics and safety status from the perspective of time–frequency and energy effectively. The following conclusions can be drawn:

(1) The proposed method can effectively suppress modal aliasing and signal noise during blasting vibration signal processing compared with the traditional EMD, WT and other related methods. Moreover, it can identify the blasting vibration characteristics of groundwater-sealed tunnel from the perspective of time–frequency and energy accurately and effectively, and provide reference basis for improving blasting excavation quality and construction safety.

(2) The cumulative average energy of blasting vibration in the frequency range of 200 Hz occupies 94.7% of the total energy and 72.5% of the total energy in the frequency range of 0–50 Hz, which is the main frequency band. Amongst the radial, tangential and vertical components, the vertical energy accounts for the largest proportion.

(3) The results of the comparison of the theoretical-actual delay time and interval of the blasting charge are consistent, verifying the feasibility of the feature extraction and analysis method proposed in this work to identify millisecond differential blasting, and that it has better resolution and identification ability for blasting vibration signal.

(4) The blasting vibration releases the largest energy at the top arch of the tunnel, and the support and safety precautions of the top arch should be strengthened. The energy release of the sidewall and the sidewall bolts are the smallest, which has little effect on the safety of the tunnel rock mass, and the vibration energy of the adjacent tunnel attenuates the fastest, but none of them exceeds the blasting safety regulations (GB6722-2014 Safety Regulations for Blasting, China).

It should be pointed out that proposed method needs to select appropriate parameters for randomness detection of MPE after CEEMDAN adaptive decomposition. Therefore, it requires multiple trials and reference literature on blasting vibration.

### Supplementary Information

Supplementary Information.

## Data Availability

All data generated or analysed during this study are included in this published article.
